# Novel transcatheter arterial embolization method for hemodynamically unstable pelvic fractures to prevent complications of gluteal necrosis

**DOI:** 10.1007/s00068-018-01066-1

**Published:** 2019-01-08

**Authors:** Takaaki Maruhashi, Fumie Kashimi, Rika Kotoh, Shun Kasahara, Hiroaki Minehara, Yuichi Kataoka, Hiroshi Nishimaki, Yasushi Asari

**Affiliations:** 1 Department of Emergency and Critical Care Medicine, Kitasato University School of Medicine, 1-15-1 Kitasato, Minami-ku, Sagamihara, Kanagawa 252-0375 Japan; 2 Department of Orthopedic Surgery, Kitasato University School of Medicine, 1-15-1 Kitasato, Minami-ku, Sagamihara, Kanagawa 252-0375 Japan; 3 Department of Cardiovascular Surgery, St. Marianna University School of Medicine, 2-16-1, Sugao, Miyamae-ku, Kawasaki, Kanagawa 216-8511 Japan

**Keywords:** Pelvic fracture, Transcatheter arterial embolization, Gluteal necrosis, Complication

## Abstract

**Purpose:**

To validate our previously designed transcatheter arterial embolization (TAE) technique for bilateral iliac arteries in unstable pelvic fractures, which is designed to also prevent gluteal necrosis and avoid vasopressors.

**Methods:**

We retrospectively analyzed the data of patients with pelvic fractures who underwent our new TAE procedure to determine the incidence of subsequent gluteal necrosis. We also compared certain variables between patients who underwent TAE before 2005 using a different technique and developed gluteal necrosis and patients who underwent TAE in 2005 and onward using our technique. Gluteal necrosis was confirmed by a radiologist based on imaging findings.

**Results:**

Seventy patients with pelvic fractures who underwent our TAE technique met the inclusion criteria (bilateral iliac arterial embolization and no embolic agent other than a gelatin sponge). Patients’ median age was 47.5 years, 33 were male, and 92.9% (65/70) had unstable fractures. Sixty-eight patients had severe multiple trauma. No patients developed gluteal necrosis following our TAE procedure and the overall survival rate was 82.9% (58/70). We found no statistically significant difference in procedure time between the previous and new technique, although the new procedure tended to be shorter. Furthermore, overall survival did not significantly differ between the groups. Multiple regression analysis revealed that TAE procedure time and external pelvic fracture fixation were independently related to gluteal necrosis.

**Conclusions:**

Our non-selective bilateral iliac arterial embolization procedure involves arresting shock quickly, resulting in no post-procedure gluteal necrosis. The procedure involves cutting the gelatin sponge rather than “pumping” and avoids the use of vasopressors.

## Background

Pelvic fractures are a relatively common fatal injury resulting from various types of trauma, including traffic accidents and falls. They cause massive retroperitoneal bleeding, and the mortality rate for patients with hemodynamic instability due to pelvic fractures remains at approximately 30% [1]. Therefore, pelvic fractures are treated by combining mechanical stabilization of the fractured region using temporary external fixation devices or external pelvic fixation, transcatheter arterial embolization (TAE), or preperitoneal pelvic packing (PPP). However, the optimal order of these treatments remains controversial. Over 80% of hemorrhage cases in pelvic fractures originate at the vein and bone surfaces [2]. It is certain that mechanical fixation of the pelvis is important because pelvic instability is likely to promote massive bleeding with movement and posture transformation. However, there are reports that about 54% of pelvic fracture patients who are hemodynamically unstable have active arterial bleeding as the cause. Thus, TAE should not be delayed due to external pelvic fixation [3]. At present, endovascular embolization of the iliac artery via TAE is the standard treatment for controlling retroperitoneal hemorrhage, particularly arterial hemorrhage, accompanying pelvic fractures [4–7]. Gluteal necrosis is a complication of pelvic fractures, and there are many reports claiming that TAE is a risk for this complication [8–10]. However, among these previously published reports, few have investigated the specifics of the TAE treatment strategies employed, such as the embolization method, embolization area, and embolic agent. Meanwhile, some reports have stated that gluteal necrosis is not related to TAE [11] or that it represents the primary injury caused by direct external force at the time of the injury becoming apparent over time [12]. Some researchers and clinicians deny that TAE itself is a risk factor for gluteal necrosis, claiming that the cause of gluteal necrosis is care during hospitalization, such as the management of infusions or the use of vasopressors. Thus, the factors that lead to gluteal necrosis associated with pelvic fractures are a topic of discussion, with no consensus having been reached.

We previously performed a preliminary study of 82 patients with pelvic fracture provided with inpatient treatment at the Kitasato University Hospital Emergency and Disaster Medical Center (hereafter referred to as “this facility”), a level 1 trauma center, between January 1997 and December 2004. There were nine cases of gluteal necrosis in the cohort, and no instances of gluteal necrosis occurred when TAE was not performed. Furthermore, we divided the 30 cases in which TAE was performed into the “no gluteal necrosis” group (21 cases) and “gluteal necrosis” group (9 cases) and compared the groups to investigate the risk factors for gluteal necrosis. As a result, direct external force (bruises, abrasions, subcutaneous hematoma, etc) to the gluteal area (*P* < 0.01), pelvic AIS (*P* < 0.01), use of vasopressors during initial treatment (*P* = 0.02), and use of a metallic coil as the embolic agent (*P* = 0.049) were factors that significantly increased the rate of gluteal necrosis [15th Japanese Society of Emergency Radiology, Fumie Kashimi, et al. (unpublished paper)]. In this preliminary study, TAE was performed in all cases of gluteal necrosis. However, we must consider that TAE, a standard treatment for severe pelvic fracture, was essential in these patients. As such, we concluded that rather than a complication caused by TAE, direct external trauma to the gluteal region caused by pelvic fracture is the biggest risk factor for gluteal necrosis. However, because embolization with a metallic coil was a risk factor for gluteal necrosis, we concluded that the TAE method used also increases the risk of gluteal necrosis.

Based on the above analysis, we decided to develop a TAE method to provide satisfactory and rapid treatment while preventing gluteal necrosis. We have been using this new method at our institution since January 2005 for the treatment of hemodynamically unstable pelvic fractures. This new method is designed to non-selectively embolize from the trunk of the bilateral internal iliac arteries, to not involve the use of vasopressors before TAE, and to make use of a gelatin sponge (GS) created by the cutting method as the embolic agent instead of a metallic coil. The goal of the present study was to verify the validity of this new TAE strategy for the treatment of hemodynamically unstable pelvic fractures.

## Materials and methods

### Study design and population

This study is a retrospective analysis of cases encountered from January 2005 to December 2015 at our institution. Pelvic fractures for which TAE was performed at our facility were treated with the previously described new TAE strategy. Patients treated with selective embolization or an embolic agent other than GS were excluded from the study.

Cases were extracted from the facility’s inpatient data bank using the keyword “pelvic fracture”. From this initial search, only cases in which TAE was performed to treat a pelvic fracture were selected. Of these, all cases that were not excluded were enrolled in the study. We extracted information regarding gluteal necrosis after admission from inpatient medical records. We did not conduct regular imaging routinely after TAE. Contrast-enhanced computed tomography (CT) or magnetic resonance imaging was performed only if gluteal necrosis was suspected from clinical symptoms or physiological findings, such as pain or skin color changes in the gluteal region and persistent fever. A definitive diagnosis of gluteal necrosis was made based on the interpretation of a radiological diagnostician.

### Initial trauma examination and pelvic fracture treatment

Initial trauma examination was performed by emergency physicians based on the Japan Advanced Trauma Evaluation and Care guidelines (JATEC™). If an unstable pelvic fracture was seen in the pelvic X-ray conducted in the primary survey, a pelvic binder was used. If the patient’s hemodynamics were unstable, a rapid transfusion of extracellular fluid was provided, and for patients who did not respond to the initial transfusion, a packed red blood cell transfusion was started. If an unstable pelvic fracture was observed on the pelvic X-ray and the patient was a non-responder (as defined above), we performed TAE using a pelvic binder. For patients who were hemodynamically stable or responders (stabilized after the initial transfusion), contrast-enhanced CT was performed as a secondary survey and, if it was determined appropriate after detailed assessment, angiography was also performed.

External pelvic fixation was performed by an orthopedic surgical specialist in patients with severe pelvic instability after TAE.

Preperitoneal pelvic packing was selected for hemodynamically unstable patients with pelvic fracture at many other facilities. There are several reports that PPP is effective for severe pelvic fractures because it can simultaneously control both arterial and venous bleeding [13–16].

For the following reasons, we have not performed PPP as much as possible at our facility. First, about half of the pelvic fracture patients are hemodynamically unstable and have arterial active bleeding as the cause. Second, the long-term use of gauze is known to cause pelvic infection. In particular, when repacking is required, the infection rate can reach 45% [16]. PPP was performed when hemodynamic instability persisted even after TAE and EF.

### TAE for pelvic fractures

In cases of pelvic fracture, angiography was considered suitable in all patients with hemodynamic instability or extravascular contrast agent leakage on contrast-enhanced CT. TAE was considered suitable if the angiography results revealed one of the following: extravascular contrast agent leakage, severe vasospasm, or vascular disruption of the major blood vessels or their branches. For TAE, endovascular treatment was performed with the participation of our facility’s full-time IVR specialist as the surgeon or primary assistant. For embolization, a 5-Fr short sheath (Medikit super sheath, Medikit co., Tokyo, Japan) was first installed in the retrograde direction into the right femoral artery. The internal iliac artery was selected using a 5-Fr cobra-type catheter (Torcon NB^®^Advantage Catheter; Cook Japan, Tokyo, Japan) or a shepherd hook-type catheter (Hanaco disposable torque catheter, Hanaco medical co., Saitama, Japan). GS was cut to a uniform size of approximately 2 × 2 mm and non-selective embolization was conducted from the origin of the internal iliac artery. As embolization was only performed on the injured side in cases of hemodynamic stability or one-sided pelvic fractures, these cases were excluded from the study. Angiography was performed once more after embolization, and embolization using the same method was repeated until it could be confirmed that any residual extravascular contrast agent leakage had disappeared. We excluded cases in which embolic agents other than GS were used at the discretion of the surgeon due to a failure to obtain hemostasis with the above methods or the presence of a marked coagulation disorder. From January 2005 to November 2013, the type of GS used was Spongel^®^ (Astellas Pharma Inc., Tokyo, Japan). From December 2013 onward, Serescue^®^ (Astellas Pharma Inc., Tokyo, Japan) was used.

### Study endpoints

The primary endpoint of this study was the rate of gluteal necrosis in patients treated with the new TAE strategy from 2005 onward. As secondary endpoints, we compared factors other than TAE strategy, such as patient background, severity of trauma [Abbreviated Injury Scale (AIS), Injury Severity Score (ISS)] (Association for the Advancement of Automotive Medicine) [17], fracture type (Arbeitsgemeinschaft für Osteosynthesefragen/Orthopedic Trauma Association class), vital signs, Shock Index, time from hospital arrival to TAE start, time required for TAE, 24-h blood transfusion volume, and outcome, between the study cases and the nine cases of gluteal necrosis that occurred before 2005.

Subsequently, multivariate analysis was performed with gluteal necrosis rate as the dependent variable to reveal the independent factors influencing the gluteal necrosis rate. The Shock Index was calculated as heart rate/systolic blood pressure and a Shock Index ≥ 1 was judged as hemorrhagic shock [18, 19].

### Statistical analysis

SPSS version 21 (IBM SPSS Statistics, Chicago, IL, USA) was used to perform all statistical analyses. Continuous and categorical variables were analyzed using the Mann–Whitney *U* test and Fisher’s exact test, respectively. In all statistical analyses, a *P* value < 0.05 was considered statistically significant. Multivariate analysis was performed using logistic regression. Independent variables were selected through likelihood ratios.

## Results

**Fig. 1:**
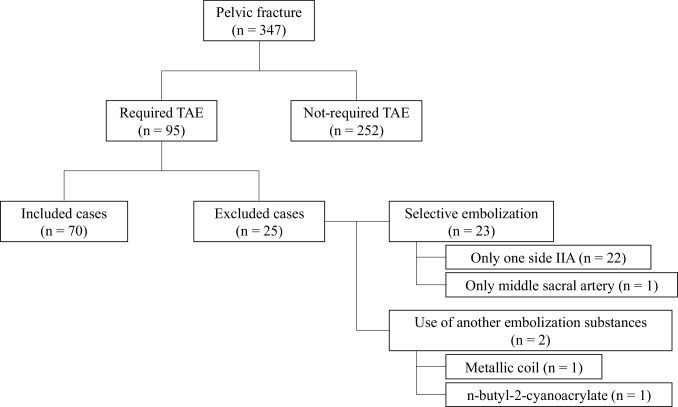
Of the 347 cases of pelvic fracture during the study period, transcatheter arterial embolization (TAE) was performed in 95. Of these, 23 cases in which selective embolization was performed (one-sided internal iliac artery embolization only in 22 cases; middle sacral artery embolization only in 1 case) and 2 cases in which an embolic agent other than gelatin sponge was used (metallic coil in 1 case; *n*-butyl-2-cyanoacrylate in 1 case) were excluded to leave a final number of 70

During the study period, 347 patients with pelvic fractures were admitted to our facility. TAE was performed in 95 (27.4%) of these patients. We excluded 23 patients who were treated with selective embolization (one-sided internal iliac artery embolization only in 22 and middle sacral artery embolization only in 1) and 2 patients treated with an embolic agent other than GS (metallic coil in 1 and *n*-butyl-2-cyanoacrylate in 1). The final number of patients was 70 (Fig. 1).

The background characteristics of the included patients are shown in Table 1. The median age was 47.5 years [interquartile range (IQR) 33.8–70] and 33 patients (47.1%) were male. Twenty patients had a Shock Index ≥ 1 at the initial examination (28.6%). According to the Arbeitsgemeinschaft für Osteosynthesefragen/Orthopedic Trauma Association classification, the pelvic fracture types were as follows: type A in 5 patients, type B in 24 patients, and type C in 41 patients. Unstable pelvic fractures (types B and C) accounted for 92.9% (65 of 70) of the cases. The median fracture AIS was 4 (IQR 4–5) and the median ISS value was 38 (IQR 19.3–4). In total, 68 patients (97.1%) had severe multiple trauma with an ISS ≥ 16. There was one case of open pelvic fracture and, with respect to complications accompanying pelvic fracture, there were three cases of bladder injury and two cases of rectal injury. In addition to TAE, external pelvic fixation was conducted in 26 cases (37.1%) and retroperitoneal gauze packing was conducted in 3 (4.3%). There were no cases in which catecholamine was used before TAE in accordance with the treatment strategy. The primary endpoint of the rate of occurrence of gluteal necrosis in study cases was 0% (0/70 cases). The overall rate of survival to discharge was 82.9% (58/70 cases).

**Table 1:** Comparison of the characteristics of the study cases and the nine patients complicated by gluteal necrosis before 2004

	2005/1–2015/12 (*n* = 70)	1997/1–2004/12 (*n* = 9)	*P* value
Age (years)	47.5 (33.8–70)	55 (23–69)	0.86
Male (%)	33 cases (47.1%)	6 cases (66.7%)	0.31
Mechanism			
Traffic accident	27 cases (38.6%)	6 cases (66.7%)	0.15
Fall	33 cases (47.1%)	1 case (11.1%)	0.07
Others	10 cases (15.3%)	2 cases (22.2%)	0.62
Vital signs at initial arrival			
Respiratory rate (breaths per minute)	24 (19.5–29.25)	30 (23–37.8)	0.05
Saturation (%)	99 (97–100)	96 (95.5–99)	0.19
Systolic blood pressure (mmHg)	104.5 (80–133.75)	76 (66–91)	0.12
Pulse rate (beats per min)	100 (77–121)	117 (74–146)	0.93
Glasgow Coma Scale (point)	13 (10–14)	15 (15–15)	0.03
Shock Index ≥ 1	20 cases (28.6%)	7 cases (77.8%)	< 0.01
Use of catecholamine before TAE	0 case (0%)	3 cases (33.3%)	< 0.01
Time from arrival to TAE (min)	70.5 (49.5–96)	79 (45–105)	0.82
Time for TAE (min)	33.5 (20.5–44.5)	70 (40–110)	0.09
Transfusion at first 24 h (unit)			
Red blood cell	10 (6–20)	11 (4.75–20)	0.88
Fresh frozen plasma	11.5 (5.5–20)	8 (5–20)	0.48
Platelet concentrate	17.5 (0–20)	20 (15–20)	0.19
Type of pelvic fracture^a^			
Type A	5 cases (7.1%)	2 cases (22.2%)	0.18
Type B	24 cases (34.3%)	2 cases (22.2%)	0.71
Type C	41 cases (58.6%)	5 cases (55.6%)	0.57
Additional treatment for pelvic fracture			
Pelvic gauze packing	3 cases (4.3%)	0 cases (0%)	0.53
External fixation	26 cases (37.1%)	6 cases (66.7%)	0.15
REBOA	0 case (0%)	2 cases (22.2%)	0.12
Open fracture (%)	1 case (1.4%)	2 cases (22.2%)	0.03
Complications associated with pelvic fracture			
Rectal injury	2 cases (2.9%)	0 cases (0%)	0.61
Bladder injury	3 cases (4.3%)	1 case (11.1%)	0.38
Morel-Lavallée lesions	3 cases (4.3%)	2 cases (22.2%)	0.16
Pelvis AIS	4 (4–5)	4 (4–5)	0.83
Max AIS of other injury site			
Head and neck	1 (0–3)	0	0.07
Chest	3 (0–4)	0 (0–3)	0.30
Abdominal	0 (0–3)	3 (0–4)	0.12
Injury severity score	38 (19.3–45)	42 (33–43)	0.99
Overall survival rate (%)	82.9%	55.6%	0.08

*TAE* transcatheter arterial embolization, *REBOA* resuscitative endovascular balloon occlusion of the aorta, *AIS* Abbreviated Injury Scale

^a^Classification of the Arbeitsgemeinschaft für Osteosynthesefragen/Orthopedic Trauma Association

Table 2 shows a comparison between the 9 of 82 patients with complicating gluteal necrosis associated with pelvic fractures in the preliminary study from January 1997 to December 2004 and the cases included in the current study. In univariate analysis of each item between the two groups, a statistically significant difference was observed for initial Glasgow Coma Scale, Shock Index ≥ 1, catecholamine use before TAE, and number of open pelvic fracture cases.

The median values for time from hospital arrival to TAE initiation in the two groups were 70.5 min (IQR 49.5–96) and 79 min (IQR 45–105). There was no statistically significant difference (*P* = 0.82). The median values for time required for TAE were 33.5 min (IQR 20.5–44.5) and 70 min (IQR 40–110). The time tended to be shorter for the new method implemented in 2005 and performed thereafter. However, this difference was not statistically significant (*P* = 0.09).

Regarding the overall survival rate, while a statistically significant difference was not observed, the rates were 82.9% vs. 55.6% (*P* = 0.08), with a higher survival rate in the new treatment strategy group. In multiple logistic regression analysis, the time required for TAE and external pelvic fixation surgery were independent factors related to the occurrence of gluteal necrosis [respectively, *P* = 0.009, odds ratio (OR) 1.030, 95% confidence interval (CI) 1.008–1.054, *P* = 0.036, OR 8.374, 95% CI 1.149–61.005].

## Discussion

The rate of gluteal necrosis following TAE for pelvic fracture varies in previous reports and lies somewhere in the range 3.3–9.4% [20, 21]. Furthermore, when complicated by gluteal necrosis, the mortality rate can reach 60%. The survival rate can be increased by preventing gluteal necrosis, prompting the need to understand the causes of gluteal necrosis in these patients.

At our institution, we have succeeded in reducing the gluteal necrosis rate to 0% through the use of a new TAE strategy, which has been performed since 2005. Multiple regression analysis revealed that other than differences in TAE strategy, the time required for TAE and whether external pelvic fixation was used are risk factors for gluteal necrosis. External fixation was considered suitable if pelvic instability was observed; this supports the hypothesis that the severity of direct force to the gluteal region rather than TAE is a primary factor for gluteal necrosis. In total, 58.6% (41/70 cases) of participants in this study had type C fractures (severe pelvic fractures). It is true that the direct force to the pelvis was not minimal in these patients. Nonetheless, in recent years, when pelvic instability is not very severe, a pelvic binder is usually used first. The fact that the number of cases in which internal fixation was used in the early stage has increased may also explain why the rate of external fixation surgery was higher in the gluteal necrosis group.

The adoption of the new strategy significantly contributed to the reduction in time required for TAE. In the new strategy, by conducting non-specific embolization from the origin of the internal iliac artery, the time required for TAE was reduced, hemodynamics stabilized quickly, and the use of vasopressors avoided. There are some pros and cons for performing embolization from the origin of the bilateral internal iliac arteries that should be discussed. Although it is commonly accepted that the intervention should be stopped at one-sided embolization or selective embolization to avoid gluteal necrosis [22, 23], some reports have stated that bilateral internal iliac artery embolization is safe, with few complications (including gluteal necrosis) [12]. Furthermore, persistent systolic blood pressure > 90 mmHg is one factor associated with the success of TAE [24]. We concluded from the previous research that bilateral internal iliac artery TAE does not represent a direct risk for gluteal necrosis, rather extreme direct force to the gluteal region and the resultant shock condition are the main causes. As such, we focused on stopping shock in the early stage through prompt TAE. The results of our study support this hypothesis. Furthermore, pelvic fractures accompanied by shock are associated with a high mortality rate [25]. Thus, it is suggested that quickly stopping shock not only prevents gluteal necrosis but also contributes to improving the survival rate.

There are several other factors to consider when assessing why the new TAE strategy adopted at our institution did not increase the rate of gluteal necrosis. First, it is possible that proximal embolism due to the GS being cut into a large (2 mm) and uniform piece encouraged the development of a collateral pathway. GS is manufactured in sheets and is usually prepared for use according to its purpose. There are two main methods of preparation: cutting and pumping. In the pumping method, the sheet-shaped product is broken into fine particles by alternatively applying pressure using two syringes and a three-way stopcock. Compared to the cutting method, more variation occurs in particle diameter when using the pumping method [26].

When embolization was performed with GS created via the pumping method, proximal embolization did not occur even though the injection site for the embolic agent was the internal iliac artery origin, rather smaller emboli accumulated in the periphery. However, when using GS created via the cutting method, by embolizing at the origin of the internal iliac artery, collateral pathways will form from the middle sacral artery, external iliac artery, or inferior mesenteric artery in the long-term. Next to consider are differences in the embolic agents used. As described above, GS induces hemostatic effects by occluding blood vessels with small-granule emboli. However, it is known to be a temporary embolic agent, with reopening usually occurring after about 3 weeks. Meanwhile, with a metallic coil, the hemostatic effects are obtained by encouraging thrombus formation by filling the blood vessel and interrupting blood flow to the areas peripheral to the coil embolization. As such, metallic coils are classified as a fundamentally permanent embolic agent. In fact, in a previous report, a metallic coil was used in four of five cases of TAE for pelvic fracture complicated by gluteal necrosis [20], suggesting that the use of a metallic coil is a risk factor for gluteal necrosis.

This study has several limitations. First, this was a single-center study and the sample size was small. In the future, it will be necessary to accumulate more case reports. Second, for the diagnosis of gluteal necrosis, imaging was conducted based on clinical symptoms. Imaging via MRI was not conducted in every case in which TAE was performed. As such, we cannot completely rule out minor gluteal necrosis complications that did not cause clinical problems. Finally, this was a retrospective study. It is possible that the quality of trauma treatment itself has improved over time or that appropriate interventions after admission, such as early stage rehabilitation or nutritional care, contributed to the reduction in the gluteal necrosis rate.

## Conclusions

The new TAE strategy used at our institution for treating hemodynamically unstable severe pelvic fracture has the following characteristics: non-selective embolization from the trunk of the bilateral internal iliac arteries, no vasopressors administered before TAE, and use of 2-mm GS created by the cutting method instead of a metallic coil as the embolic material. This new strategy does not increase the risk of gluteal necrosis.
